# Electrophysiological Correlates of the Interaction of Physical and Numerical Size in Symbolic Number Processing: Insights from a Novel Go/Nogo Numerical Stroop Task

**DOI:** 10.3390/brainsci13050702

**Published:** 2023-04-22

**Authors:** Judit Pekár, Wiebke Hofmann, Balázs Knakker, Sascha Tamm, Annette Kinder

**Affiliations:** 1 Institute of Psychology of Learning, Department of Education and Psychology, Freie Universität Berlin, 14195 Berlin, Germany; 2 Grastyán Translational Research Center, University of Pécs, 7624 Pécs, Hungary; 3 Institute of Experimental and Cognitive Neuropsychology, Department of Education and Psychology, Freie Universität Berlin, 14195 Berlin, Germany

**Keywords:** numerical Stroop task, go/nogo task, N2, P3, facilitation

## Abstract

The interaction of physical and numerical size has been investigated and repeatedly demonstrated in the numerical Stroop task, in which participants compare digits of different physical sizes. It is, however, not entirely clear yet what psychological processes contribute to this interaction. The aim of the present study is to investigate the role of inhibition in the interaction of physical and numerical size, by introducing a novel paradigm that is suitable to elicit inhibition-related event-related potential components. To this end, we combined the go/nogo paradigm with the numerical Stroop task while measuring EEG and reaction times. Participants were presented with Arabic number pairs and had to press a button if the number on one side was numerically larger and they had to refrain from responding if the number on the other side was numerically larger. The physical size of the number pairs was also manipulated, in order to create congruent, neutral, and incongruent trials. Behavioural results confirmed the well-established numerical distance and numerical Stroop effects. Analysis of electrophysiological data revealed the classical go/nogo electrophysiological effects with numerical stimuli, and showed that peak amplitudes were larger for nogo than for go trials on the N2, as well as on the P3 component, on frontal and midline electrodes. When analysing the congruency effects, the peak amplitude of N2 was larger in incongruent trials than in neutral and congruent trials, while there was no evidence of a congruency effect on the P3 component peaks. Further analysis of the electrophysiological data revealed an additional facilitatory effect in the go trials, as well as an additional interference effect in the nogo trials. Taken together, it seems that interference effects are probably resolved by inhibitory processes and that facilitatory effects are affected by different cognitive control processes required by go versus nogo trials.

## 1. Introduction

A central question in research on numerical cognition is how numerical information and continuous magnitudes, such as physical size, interact. Key to finding answers to this question is the numerical Stroop task, in which participants are presented with pairs of Arabic digits and their task is to decide which number is larger, based either on their numerical size or on their physical size [1]. According to these two dimensions the number pairs may be congruent (the numerically larger number is also physically larger) or they may be incongruent (the numerically larger number is presented in smaller physical size). Besides the well-known numerical distance effects—i.e., shorter reaction times for large compared to small numerical distances—studies using this paradigm have repeatedly reported facilitation and interference effects, which show that not only task-relevant information but also task-irrelevant information is processed [1,2,3,4,5,6,7,8,9,10,11,12]. Thus, the paradigm has been used to investigate the cognitive processes that are involved in the interaction of task-relevant and task-irrelevant stimulus features in general. Furthermore, it has also been used in the narrower context of numerical research, in order to investigate at what stage of processing the interaction of physical and numerical information occurs, and related to this, whether numbers and other magnitudes are processed by a common magnitude system or by separate systems.

Two competing hypotheses have been formulated that aim to explain the interaction of numerical and non-numerical information in the numerical Stroop task. According to the early interaction account, numerical and physical dimensions of the stimuli are processed by common neural representations in the intraparietal sulcus and interact at an “early” processing stage before the appropriate motor response is selected, prepared, and executed. In contrast, the late interaction account suggests that numerical and size information are processed in parallel by different neural substrates and the conflict occurs at a later stage upon response initiation in the motor cortex [2,4,9,13]. Thus, an early interaction would support the idea that numerical and size information is subserved by shared neural substrates, while a late interaction would indicate distinct neural substrates. To differentiate between these two opposing accounts, researchers search for the locus of congruency effects in the numerical Stroop task, while measuring EEG and comparing certain event-related potential components (ERPs), such as the P3 and the lateralised readiness potential [2,4]. The P3 component is a positive deflection which is usually observed over centro-parietal electrode sites 300–500 ms after stimulus presentation, and it reflects stimulus evaluation and categorisation processes [2,14,15,16]. Thus, congruency effects on the P3 indicate that the interaction of numerical and size information occurs at the stage of stimulus processing (early interaction). In contrast, the lateralised readiness potential (LRP) reflects selective motor activation which is larger over electrode sites contralateral to the response hand. It is calculated by subtracting ipsilateral activity from contralateral activity. Congruency effects on the LRP suggest that the interaction occurs at the response level (late interaction).

Using these neural markers, Gebuis et al. [2] investigated whether the interaction of numerical information and continuous magnitudes occurs at the motor response stage or rather prior to that, during processes which lead up to stimulus evaluation and categorisation. They examined the peak latency and the peak amplitude of the P3 component, together with stimulus- and response-locked LRPs. The P3 peak appeared later for trials with small compared to large numerical distances, as well as for incongruent trials compared to congruent trials. The amplitude of the P3 component was larger for large numerical distances and for congruent trials. Furthermore, they found congruency effects on the stimulus-locked but not on the response-locked lateralised readiness potentials. Taken together, their results suggest that physical size interacted with numerical size before the end of stimulus evaluation and before the preparation or initiation of a response started. This pattern of findings is in line with the early interaction account and a general magnitude system. Kadosh et al. [4] found the same pattern of congruency effects on the P3 amplitude as Gebuis et al. [2]. However, they [4] also reported late interaction effects on the response-locked LRPs, which suggests that the processing of numerical and size information may under certain circumstances be subserved by distinct rather than shared neural substrates. Consequently, the matter of early versus late interaction of numerical and physical size has not been entirely settled yet. Nevertheless, it is clear that studies using the numerical Stroop paradigm have repeatedly confirmed that numerical and physical size interact and that the interaction may occur either at the perceptual or at the motor level.

Even though numerous studies have investigated the locus of the interaction between numerical and physical size information in the numerical Stroop task, to the best of our knowledge, none of them investigated the exact nature of the psychological processes that underlie this interaction. Thus, the question arises: What kind of psychological processes are involved in the behavioural and electrophysiological effects that are observed in the numerical Stroop task? A process that may underlie the influence of physical size on Arabic number processing is inhibition. For example, it is possible that in the numerical Stroop task the physical size of the number pairs is inhibited, in order to ensure responding to numerical size. In line with this idea, executive functions, especially inhibition, have been reported as an important factor in numerical processing and development. Even though a large number of studies have investigated the relationship between inhibition and numerical processing [17,18,19], a common feature of them is that they usually rely on behavioural methods [20].

Inhibition, however, is not a unitary but rather a multifaceted construct, and recent studies have focused on disentangling the electrophysiological signatures of different types of inhibition [21,22,23,24,25]. Related to this, a major question in inhibition research is how different types of inhibition contribute to various behavioural and developmental profiles. One of the most common methods to investigate the different types of inhibitory processes is administering the go/nogo task while measuring EEG. In this task, participants are presented with a stream of very simple stimuli (e.g., the letters M and W), and they are asked to respond with a button press to frequent go trials (e.g., M) and refrain from responding to infrequent nogo trials (e.g., W). The electrophysiological correlates of inhibitory control that are measured in the go/nogo task are the N2 and P3 ERP components. The N2 ERP component is a fronto-central, stimulus-locked, negative deflection occurring 200–350 ms post-stimulus, that is larger in nogo than in go trials [23,26,27,28,29]. By contrast, the P3 component is a positive deflection, often found between 300 and 500 ms after stimulus presentation but—as opposed to the previously mentioned categorisation-related P3 component—the P3 in inhibition tasks has a more anterior topography, that is referred to as the nogo anteriorisation (NGA). The nogo anteriorisation is thought to reflect activation in premotor areas, inferior frontal cortex, and in the cingulate region which led to the conclusion that the component reflects response inhibition [30,31,32,33,34,35,36,37,38,39,40,41]. Because in the go/nogo task both the N2 and P3 components are enhanced in nogo compared to go trials, originally both components were considered as a marker of response inhibition.

Studies which focus on disentangling the electrophysiological signatures of different types of inhibition usually include the go/nogo and Flanker tasks, as well as their modified or hybrid versions [21,22,23,25]. In Flanker tasks, for example, participants are presented with five arrows and their task is to respond to the central target arrow, while the flanking arrows may be congruent or incongruent with the central target arrow (e.g., ⟶⟶⟶⟶⟶ and ⟶⟶⟵⟶⟶, respectively). In the hybrid version of this task, the arrows are further manipulated in a way that they either require a motor response (go trials—e.g., arrows point to left/right) or they require refraining from responding (nogo trials—e.g., arrows point up/down). Such a trial arrangement allows the separation of two types of inhibition and their electrophysiological signatures: nogo trials require response inhibition while incongruent trials require resisting interference from the flanking arrows. Groom and Cragg [23] implemented such a hybrid task and found that the N2 was enhanced in incongruent compared to congruent trials, while the P3 was larger in trials that required response inhibition. Similar findings were reported by Xie et al. [25], who found that interference inhibition was associated with larger N2 negativities, while trials requiring response inhibition induced larger positivity on the P3. In sum, the findings support the notion that response inhibition induced by nogo trials, and response conflict induced by incongruent trials, have differential effects on the N2 and P3 ERP components.

At this point, it is important to note, that various terms have been used to characterise the different types of inhibitory processes, e.g., response conflict and response inhibition [23], interference suppression and response inhibition [21,22,24], stimulus interference control and response interference control [42], interference inhibition and response inhibition [25]. Similarly to Xie et al. [25], we use the terms interference inhibition and response inhibition. Here, interference inhibition refers to resisting interference from irrelevant or misleading information, while response inhibition refers to stopping a prepotent response. For example, if inhibition is implicated in the numerical Stroop task, then analogously to the previously described early and late interaction accounts, it is possible that inhibition occurs at an “early stage”, meaning that interference from irrelevant physical size is inhibited presumably at the stage of perceptual processing. Another possibility is that inhibition occurs at a “later stage”, meaning that a prepotent response induced by physical size must be inhibited. Similar interpretations have been made by Soltész et al. [7], who compared the electrophysiological correlates of the congruency effects in the numerical Stroop task between children and adults. They reported similar facilitation effects on the P3 component in both groups but found a larger interference effect in children than in adults. They concluded that, because children have less well-developed response control and executive functions than adults, the larger interference effects are due to less efficient inhibition of irrelevant physical size information. Even though this interpretation seems plausible, it is important to note that it is based on modulations of the P3 component which—as described earlier—in this context reflects stimulus evaluation and categorisation processes and not cognitive control functions per se. Thus, it was not possible to directly assess the involvement of inhibition in the interaction of physical and numerical size in that study.

As mentioned before, numerous studies have investigated the relationship between numerical processing and inhibition. However, a common feature of them is that they usually rely on behavioural methods [20]. For example, inhibition is measured in one task and then numerical cognition in another, then the relationship between the performance on these two kinds of tasks is measured. These methods deliver important insights on the relationship between inhibition and numerical abilities but they are limited in terms of investigating the contribution of the different types of inhibitions and assessing whether such inhibitory processes are directly linked to the well-documented interaction of physical and numerical size in numerical Stroop tasks. On the one hand, there are a handful of papers that implement the numerical Stroop task to investigate the electrophysiological correlates of inhibition, however their major intent does not lie in answering questions about numerical processing per se, but rather in deciphering the developmental profiles or in elucidating the functional definition of certain neural markers [10,43]. On the other hand, ERP studies that investigated the locus of interaction between physical and numerical size in the numerical Stroop task did not address whether inhibition underlies this interaction [2,3,4,7,9,12].

Interestingly, even though cognitive control functions, especially inhibition, have been implicated in numerical processing, and the N2 component has been associated with cognitive control, or more specifically, with suppressing interference from irrelevant stimulus features, only a handful of papers have investigated how the N2 component is modulated by congruency in the numerical Stroop task. For example, Huang et al. [3] asked participants to judge number pairs based either on their physical size (which number is physically larger?—size task) or on their numerical size (which number is numerically larger?—number task). Interestingly, in the size task they found a facilitation effect on the N2 component, i.e., less negative N2 amplitude in congruent compared to neutral and incongruent trials. In the number task, however, they did not report any congruency effects on the N2 component. Another study, by Yao et al. [12], investigated the effects of long-term abacus-based mental training on children’s numerical processing. They found that two years later, children who received the training showed congruency effects on the N2 as well as on the P3 components, while children of the control group showed congruency effects only on the P3. The authors concluded that abacus-based mental training strengthens the relationship between number symbols and magnitude representation, which in turn leads to faster and more automatic numerical processing. In sum, not only are the number of studies that investigate the N2 in numerical Stroop scarce, but also their findings are inconclusive.

In the present study, we aim to fill in the aforementioned gaps and explicitly test whether inhibition underlies the interaction of physical size and numerical size in the numerical Stroop task by examining related electrophysiological correlates. Because the N2 and P3 components are reliably elicited and because they have been shown to reflect interference inhibition and response inhibition in go/nogo as well as in hybrid tasks, we also created a hybrid paradigm and combined the go/nogo task with the numerical Stroop task. We presented participants with congruent, neutral and incongruent Arabic number pairs and instructed them to press a button if the number on one side was numerically larger and refrain from responding if the number on the other side was numerically larger, while we measured their EEG, accuracy, and reaction times. Creating such a hybrid task has three advantages. One, including a go/nogo manipulation into the task makes it possible to obtain an unconfounded measure of response inhibition. The original numerical Stroop task is a classical two-choice task, where participants always have to implement one overt motor response. Therefore, inhibition of one response is always confounded with the initiation of the other. An unconfounded measure of response inhibition can be obtained when participants have to choose between withholding the motor response and implementing a certain motor response but not choosing between two possible motor responses. Two, combining the go/nogo paradigm with the numerical Stroop task allows us to directly address the role of inhibition and differentiate between interference inhibition and response inhibition in the interaction of physical and numerical information, while the inhibition-related N2 and P3 ERP components can be measured. Furthermore, it also provides insights about the processing stage at which inhibition may occur in numerical processing. Three, including not only congruent and incongruent, but also neutral trials, makes it possible to separately examine the interference and facilitation effects on the ERP components. This is not possible in the classical inhibition tasks, e.g., in the Flanker task, because those usually include only congruent and incongruent trials but no neutral trials.

In order to assess whether implementing this novel hybrid paradigm allows the elicitation of the inhibition-related N2 and P3 components, first we contrasted go and nogo trials of large numerical distances (small numerical distances were always go trials, for more details and reasoning see Materials and Methods section). In accordance with the classical go/nogo tasks, we expected to observe larger negativities on the N2 component over fronto-central electrode sites for nogo compared to go trials, and we expected larger positivities on the P3 components over centro-parietal electrodes for nogo compared to go trials. Second, we also analysed congruency effects on these components to investigate how physical size manipulations modulate the N2 and/or P3 components in this task. Third, in order to assess whether the repeatedly reported interference and facilitation effects of the numerical Stroop task can also be observed in this novel hybrid paradigm, we performed a mass univariate analysis to determine possible onset and offset latencies, to assess at what point in time incongruent trials differ from neutral trials (interference) and congruent trials differ from neutral trials (facilitation). Additionally, we also investigated whether we could extend the findings by Gebuis et al. [2] and Kadosh et al. [4] with regards to the categorisation-related P3 peak latency and amplitude and find the numerical distance and congruency effects on the categorisation-related P3 component. To this end, we compared peak latency and peak amplitude between small and large numerical distances and different congruency conditions in go trials and expected equivalent results to those of Gebuis et al. [2] and Kadosh et al. [4].

## 2. Materials and Methods

### 2.1. Participants

In total 23 individuals were included in the study (ten females, age: M = 24.91, SD = 3.93, range: 18–34 years), of which three were excluded due to technical problems occurring during data acquisition. All of the individuals were right-handed, had normal or corrected-to-normal vision and did not report any psychiatric or neurological disorder. They gave written informed consent and received either course credit or monetary compensation for participation. The experimental procedure was approved by the local ethics committee of the Free University Berlin. Data from four participants were not included in the data analysis because more than 25% of their EEG data contained artefacts. Thus, the final dataset consisted of data from 16 participants (seven females, age: M = 23.81, SD = 3.40, range: 18–34 years).

### 2.2. Apparatus, Stimuli, and Procedure

**Figure 1:**
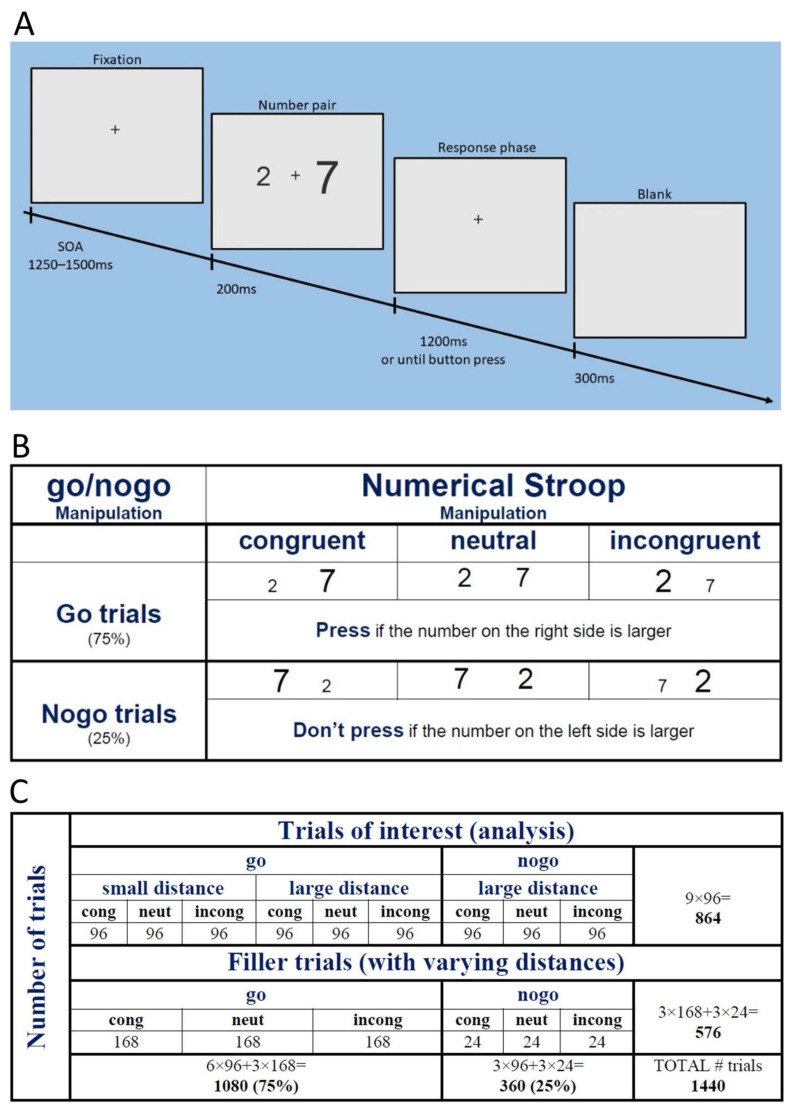
(**A**) Experimental design with timing information. (**B**) Example trials for each response type and congruency manipulation. (**C**) Number of trials by trial type in the experiment. The trials of interest always had a distance of one or five, while filler trials could have all distances between one and nine.

Trials were presented on a 23-inch monitor using PsychoPy [44,45]. Participants were presented with pairs of Arabic digits ranging from 1 to 9 and their task was to indicate whether the number on the left side or the number on the right side was numerically larger. We manipulated (1) the response type, in order to create go and nogo trials, (2) the physical size of the numbers, in order to create different congruency conditions, as well as (3) the numerical distance between the numbers, in order to include small and large numerical distances. In terms of response type manipulation, participants either had to press a button if the number on one side was numerically larger (go trials) or they actively had to refrain from responding if the number on the other side was numerically larger (nogo trials, also see Figure 1B). In the entire experiment the ratio of go versus nogo trials was 0.75 and 0.25, respectively. All participants were instructed to press the right CTRL button with their right index finger in go trials. The side-to-response assignment was counterbalanced across the participants. As a result, the final dataset included 9 participants who responded with button press when the number on the left side was larger (no response otherwise) and 7 participants who had to push a button when the number on the right side was numerically larger (no response otherwise). Congruency conditions were created by manipulating the physical size of the numbers and presenting them in various font sizes (Figure 1B): in congruent trials the numerically larger number had a larger font size than the numerically smaller number. In incongruent trials the numerically larger number was presented in smaller font size, whereas in neutral trials both numbers were presented in the same font size. For the numerical distance manipulation, we created all available number pairs with a distance of one for the small distance condition (e.g., 1 2, 2 3, 3 4, and so on) and with a distance of five for the large distance condition (e.g., 1 6, 2 7, 3 8, and so on). These number pairs constituted the trials of interest and were included in the behavioural and EEG data analysis. For the analysis of go versus nogo, only large distance trials were included. These trials occurred equally often throughout the experiment. The number of congruent, neutral, and incongruent trials was also equal, both during the entire experiment as well as in the analysis. We also included filler trials in the task in order to (1) avoid expectation effects (e.g., trials with a distance of five are always nogo trials), and (2) to keep the ratio of go and nogo trials at 0.75/0.25 throughout the experiment while also keeping the number of trials that were included in the analysis equal. Filler trials could have all possible distances between 1 and 9 and they could be all possible combinations of go/nogo and congruency manipulations (Figure 1C). The viewing distance was about 65 cm. The stimuli were presented with a width of 0.8 and a height of 1.15 in degrees of visual angle for small, 1.32 and 1.94 degrees for large, as well as with 0.97 and 1.5 degrees for neutral trials.

The experiment started with detailed instructions and eight practice trials with feedback. After the practice trials, no feedback was given to the participants. Each trial began with a fixation cross presented for 1250–1500 ms, with a stimulus onset asynchrony of at least 10 ms. This was followed by a number pair displayed for 200 ms and a fixation cross. The fixation cross was shown for a maximum of 1250 ms or until the participant pressed a button. In either case, a blank screen was presented for 300 ms before the next trial started (Figure 1A).

Participants completed six experimental blocks, each consisting of 240 trials (1440 trials in total). Each block contained 144 trials of interest and 96 filler trials. The ratio of go/nogo trials was 0.75/0.25. Congruent, neutral, and incongruent trials were presented equally often. This arrangement resulted in 96 trials of interest for each response type (go versus nogo), for each distance (small versus large), as well as for each congruency condition (congruent, neutral, incongruent) (Figure 1C).

### 2.3. Recording and Preprocessing of Electrophysiological Data

Electrophysiological data was recorded from 64 active electrodes placed according to the extended international 10–10 system (actiCAP system, BrainProducts, Munich, Germany). All electrodes were online referenced to the FCz, while AFz served as the ground electrode. Impedances were kept below 10 kΩ for the reference and ground electrodes and below 20 kΩ for the active electrodes. The recordings were amplified using the BrainAmp system (BrainProducts, Munich, Germany). The sampling rate was 500 Hz.

EEG data was preprocessed using EEGlab [46]. The data was filtered using a low-pass filter of 20 Hz, a high-pass filter of 0.1 Hz, and a notch filter of 50 Hz (each 24 dB/oct). Technical artefacts were removed manually and bad channels were interpolated using spherical lines before carrying out independent component analysis to remove ocular artefacts from the EEG signal. The EEG signals were re-referenced to the average of all included electrodes and then segmented into epochs, from 200 ms prior to 800 ms after the stimulus presentation. The 200 ms pre-stimulus interval was used for the baseline correction. Trials with artefacts and filler trials were removed from the data analysis. Artefactual trials were detected using EEGlab’s moving window peak-to-peak method, with a window width of 200 ms, a window jump of 50 ms, and a threshold of 75 µV. Average ERPs were conducted for each participant, trial type, and electrode.

Based on previous literature that investigated the N2 and P3 components in (hybrid) go/nogo paradigms, we expected effects on the midline electrodes [21,22,23,26,27]. Visual inspection confirmed our expectations, thus, the N2 event-related potential was measured as the maximum negative amplitude between 250 and 350 ms on electrodes Fz, FCz, and Cz. The inhibition-related P3 component was defined as the maximum positive amplitude between 400 and 600 ms on the fronto-central and centro-parietal electrode sites Fz, FCz, Cz, and CPz. We also measured the categorisation-related P3 component, which was defined according to Gebuis et al. [2], and was calculated as the peak amplitude and the peak latency between 300 and 800 ms on electrode Pz.

## 3. Results

### 3.1. Behavioural Results

#### 3.1.1. Reaction Time—Distance Effects in Go Trials

**Figure 2:**
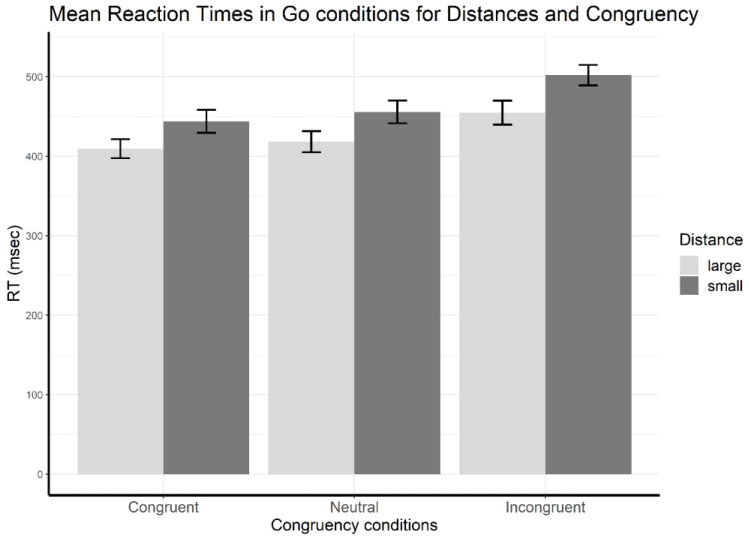
Reaction time results—mean reaction times in go trials for each distance and congruency condition. Error bars indicate standard error of the mean.

The two-way repeated measures ANOVA showed a main effect of distance (*F*(1,15) = 154.67, *p* < 0.001, $\eta_{p}^{2}$ = 0.91) and a main effect of congruency (*F*(2,14) = 190.05, *p* < 0.001, $\eta_{p}^{2}$ = 0.96) (Figure 2). The main effect of distance was a result of faster responses for large (*M* = 427.51, *SD* = 13.33) compared to small numerical distances (*M* = 467.19, *SD* = 13.74). Follow-up tests on the main effect of congruency revealed shorter reaction times for congruent than for neutral (*t*(15) = −4.30, *p* < 0.001, *Cohen’s d* = −1.08, congruent: *M* = 426.69, *SD* = 13.13, neutral: *M* = 437.02, *SD* = 13.65) and shorter reaction times for congruent than for incongruent trials (*t*(15) = −19.31, *p* < 0.001, *Cohen’s d* = −4.83, incongruent: *M* = 478.33, *SD* = 13.78), as well as shorter reaction times for neutral than for incongruent trials (*t*(15) = −16.02, *p* < 0.001, *Cohen’s d* = −4.01).

#### 3.1.2. Accuracy Data—Go/Nogo Effects

**Figure 3:**
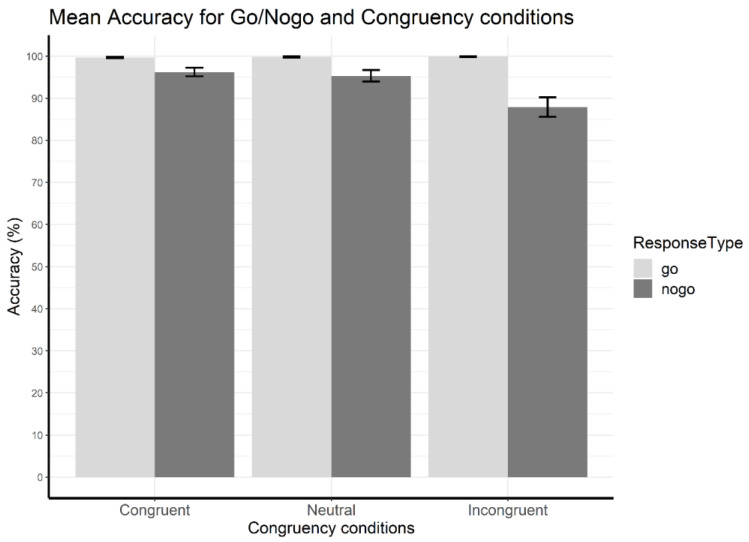
Accuracy results—mean accuracy for each go/nogo and congruency condition. Error bars indicate standard error of the mean.

The two-way analysis of variance on accuracy data revealed a main effect of go/nogo (*F*(1,15) = 22.57, *p* < 0.001, $\eta_{p}^{2}$ = 0.60), a main effect of congruency (*F*(1.21,18.15) = 24.10, *p* < 0.001, $\eta_{p}^{2}$ = 0.62), and an interaction of go/nogo and congruency (*F*(1.17,17.59) = 22.89, *p* < 0.001, $\eta_{p}^{2}$ = 0.60) (Figure 3). The interaction was followed-up by two one-way ANOVAs, conducted on factor congruency separately for the go and nogo trials. They revealed that the congruency effect was absent for the go trials (*F*(2,30) = 1.0, *p* > 0.05, $\eta_{p}^{2}$ = 0.06) but present for the nogo trials (*F*(2,30) = 23.71, *p* < 0.001, $\eta_{p}^{2}$ = 0.61). Pair-wise tests on the main effect of congruency for nogo trials showed that performance in incongruent trials (*M* = 87.89, *SD* = 9.19) was significantly lower than in congruent (*t*(15) = 4.81, *p* < 0.001, *Cohen’s d* = 1.20, *M* = 96.22, *SD* = 4.04) and neutral trials (*t*(15) = 5.51, *p* < 0.001, *Cohen’s d* = 1.38, *M* = 95.31, *SD* = 5.55), whereas the latter two did not differ from each other (*t*(15) = 1.33, *p* > 0.05, *Cohen’s d* = 0.33). The main effect of go/nogo was due to significantly higher accuracy in go trials (*M* = 99.78, *SD* = 0.36) than in nogo trials (*M* = 93.14, *SD* = 5.87), and follow-up *t*-tests showed that this effect was present for all congruency conditions (congruent: *t*(15) = 3.90, *p* < 0.05, *Cohen’s d* = 0.97; neutral: *t*(15) = 3.40, *p* < 0.05, *Cohen’s d* = 0.85; incongruent: *t*(15) = 5.24, *p* < 0.001, *Cohen’s d* = 1.31).

### 3.2. Event-Related Potentials: Peak Analysis

#### 3.2.1. Modulation of the Inhibition-Related N2 by Trial Type

**Figure 4:**
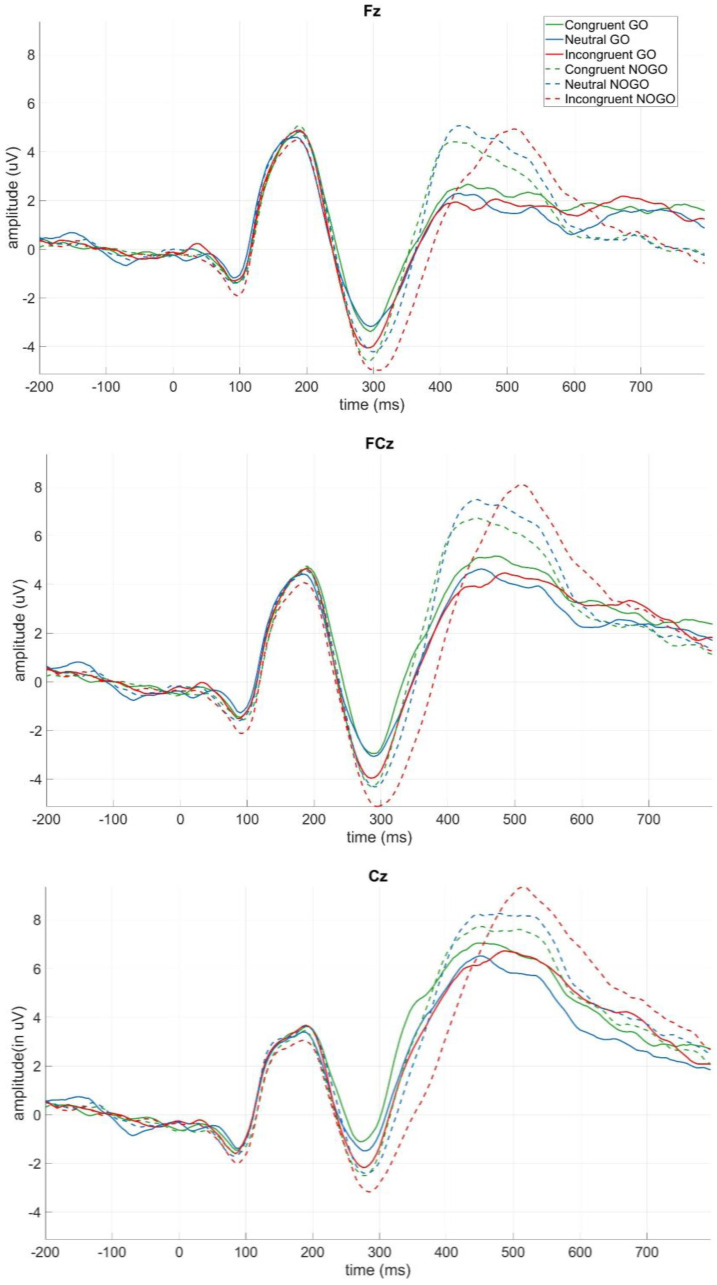

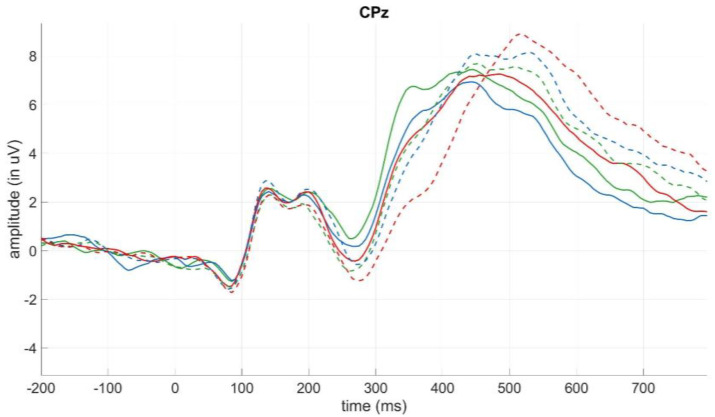
Go/nogo and congruency effects on the peak amplitude of the inhibition-related N2 and P3 components on electrodes Fz, FCz, Cz, and CPz. Positive up.

The three-way ANOVA revealed main effects of go/nogo (*F*(1,15) = 10.47, *p* < 0.05, $\eta_{p}^{2}$ = 0.41), congruency (*F*(2,30) = 6.13, *p* < 0.05, $\eta_{p}^{2}$ = 0.29), and electrode (*F*(2,30) = 24.42, *p* < 0.001, $\eta_{p}^{2}$ = 0.62) (Figure 4). None of the interactions were significant. The main effect of go/nogo was due to larger negative amplitudes in nogo than in go trials (*M* = −5.25, *SD* = 3.89 and *M* = −3.69, *SD* = 2.91, respectively), whereas the congruency main effect reflected significantly larger negative peak amplitudes in incongruent trials than in congruent (*t*(15) = 3.12, *p* < 0.05, *Cohen’s d* = 0.78, incongruent: *M* = −5.07, *SD* = 3.37, congruent: *M* = −4.12, *SD* = 3.32), as well as in neutral trials (*t*(15) = 3.12, *p* < 0.05, *Cohen’s d* = 0.78, neutral: *M* = −4.23, *SD* = 3.41). Congruent and neutral trials did not differ from one another (*t*(15) = 0.32, *p* > 0.05, *Cohen’s d* = 0.08). The main effect of electrode was caused by significantly less negative peak amplitudes on electrode Cz than on electrodes Fz (*t*(15) = −5.69, *p* < 0.001, *Cohen’s d* = −1.42, Cz: *M* = −3.03, *SD* = 3:12, Fz: *M* = −5.24, *SD* = 3.22) and FCz (*t*(15) = −5.87, *p* < 0.001, *Cohen’s d* = −1.47, FCz: *M* = −5.15, *SD* = 3.82). Electrodes Fz and FCz did not differ from each other (*t*(15) = 0.79, *p* > 0.05, *Cohen’s d* = −0.07).

#### 3.2.2. Modulation of the Inhibition-Related P3 by Trial Type

The three-way ANOVA resulted in a main effect of go/nogo (*F*(1,15) = 22.03, *p* < 0.001, $\eta_{p}^{2}$ = 0.60), and a main effect of electrode (*F*(1.53,22.89) = 31.13, *p* < 0.001, $\eta_{p}^{2}$ = 0.68) (Figure 4). The main effect of congruency did not reach significance (*F*(2,30) = 0.53, *p* > 0.05, $\eta_{p}^{2}$ = 0.04). The main effect of go/nogo was due to significantly larger positive amplitudes in nogo trials than in go trials (*M* = 8.68, *SD* = 2.74 and *M* = 6.56, *SD* = 3.48, respectively). A post hoc test on the electrode main effect revealed that peaks were significantly more positive on electrode Cz (*M* = 8.99, *SD* = 3.50) than on Fz (*M* = 5.0, *SD* = 2.92, *t*(15) = −6.55, *p* < 0.001, *Cohen’s d* = −1.64) and FCz (*M* = 7.71, *SD* = 3.29, *t*(15) = −5.34, *p* < 0.001, *Cohen’s d* = −1.3334), and also on CPz (*M* = 8.79, *SD* = 3.09), again compared to Fz and FCz (*t*(15) = −5.8884, *p* < 0.001, *Cohen’s d* = −1.46 and *t*(15) = −2.64, *p* < 0.05, *Cohen’s d* = -0.66, respectively). These latter two also differed significantly with peaks significantly more positive on FCz than on Fz (*t*(15) = −5.87, *p* < 0.001, *Cohen’s d* = −1.47).

#### 3.2.3. Modulation of the Categorisation-Related P3 by Trial Type

**Figure 5:**
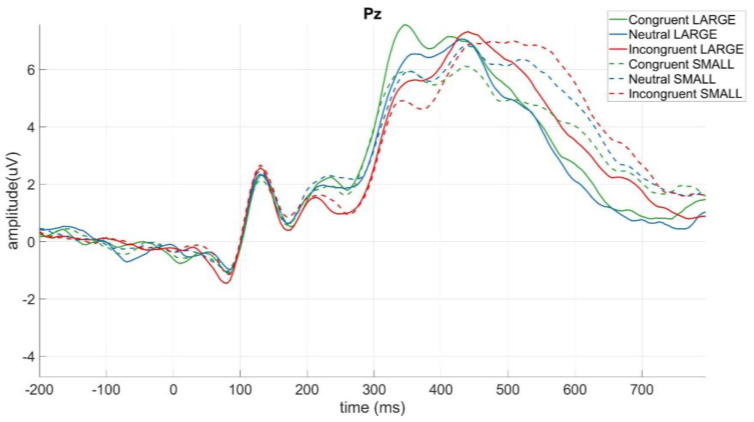
Effect of numerical distance and congruency on the peak latency of the categorisation-related P3 component on electrode Pz.

The two-way analysis of variance on the P3 peak latency (go trials) revealed significant main effects of distance (*F*(1,15) = 11.43, *p* < 0.05, $\eta_{p}^{2}$ = 0.43) and congruency (*F*(2,30) = 6.57, *p* < 0.05, $\eta_{p}^{2}$ = 0.30) (Figure 5). The main effect of distance was a result of the peak appearing later for small numerical distances (*M* = 452.60, *SD* = 92.67) than for large numerical distances (*M* = 421.98, *SD* = 66.44). The main effect of congruency was due to peaks appearing earlier for congruent trials (*M* = 411.88, *SD* = 64.09) than for neutral (*M* = 435.47, *SD* = 96.37) and for incongruent trials (*M* = 464.53, *SD* = 92.28). Post hoc analysis revealed that this difference was significant between congruent and incongruent trials (*t*(15) = −3.17, *p* < 0.05, *Cohen’s d* = −0.79). The two-way analysis of variance on peak amplitude did not reveal any significant differences.

### 3.3. Event-Related Potentials: Mass Univariate Analysis

Permutation-based test analysis on difference waves in the go condition showed that congruent and neutral trials started to show differences on electrode CPz from 325 ms to 350 ms (critical t-scores: +/−4.25, that corresponds to a test-wise alpha level of 0.000706, total number of comparisons 484, total number of permutations 2500). No significant differences were detected between incongruent and neutral trials.

In contrast, in nogo trials significant differences were detected between incongruent and neutral trials, starting at 360 ms on the Fz electrode, which then spread to all other midline electrodes and lasted up to 420 ms on Cz and CPz (critical t-scores: +/−4.16, that corresponds to test-wise alpha level of 0.000832). In nogo trials, however, no significant differences between neutral and congruent trials were detected.

## 4. Discussion

The aim of the present study was to investigate the role of inhibition in the interaction of numerical and physical size in the numerical Stroop task by examining related electrophysiological components. To this end, we introduced a novel hybrid paradigm and combined the numerical Stroop task and the go/nogo task. We presented participants with congruent, neutral, and incongruent Arabic number pairs and asked them to press a button if the number on one side of the screen was numerically larger and to refrain from responding if the number on the other side of the screen was numerically larger [1,26,27]. This arrangement allowed us to measure the well-established inhibition-related N2 and P3 ERP components. Analysis of reaction time and accuracy data confirmed the standard behavioural effects reported by previous studies that implemented the numerical Stroop task or the go/nogo task. Participants responded faster and made fewer errors on large compared to small numerical distances, as well as in congruent compared to neutral and incongruent trials. As for the go/nogo manipulation, participants generally made fewer errors in go than in nogo trials, and in the case of nogo trials a congruency effect was also observed. Fewer errors were made in congruent than in neutral and in incongruent trials.

Beyond replicating basic behavioural effects, we had four objectives. First, we were interested in whether this hybrid paradigm is suitable to elicit the classical go/nogo effects on the N2 and P3 ERP components. Second, by analysing the congruency effects on the inhibition-related N2 and P3 components, we wanted to investigate how physical size manipulations modulate the N2 and P3 components in this task. Third, in order to assess whether the repeatedly reported facilitation and interference effects of the numerical Stroop task can be observed in this hybrid paradigm, we implemented a mass univariate analysis and contrasted neutral trials with congruent and incongruent ones separately in go and nogo trials. Finally, we also examined whether we could extend the findings of Gebuis et al. [2] and Kadosh et al. [4], who reported congruency and distance effects on the amplitude and the peak latency of the categorisation-related P3 component.

When looking at the effects of the go/nogo manipulation on the N2 and P3 ERP components, we found that nogo trials elicited more negative N2 as well as more positive P3 components than go trials. These electrophysiological findings are in accord with those observed in classical go/nogo tasks and hybrid tasks [28,29,30,39,47,48,49,50]. Thus, this study is the first one to show this effect on the N2 and P3 components in a hybrid go/nogo numerical Stroop task. Furthermore, it shows that this novel hybrid paradigm is suitable to elicit these inhibition-related ERP components and therefore to investigate whether and how inhibitory processes underlie the interaction of physical and numerical size. As opposed to classical inhibition tasks, which can be described as simple decision tasks (e.g., respond when letter M is presented but do not respond when the letter W is presented), in the current paradigm, numerical judgements were made, as participants had to decide which of the two presented numbers was numerically larger. Thus, the reported differences between go and nogo trials on the N2 and P3 components also show that implementing certain modifications to the go/nogo task is an effective tool to investigate inhibition in more complex cognitive processes as well.

When analysing the interaction of physical and numerical size in go and nogo trials, we found that congruency effects occurred on the N2 but not on the P3 component. Incongruent trials were more negative than congruent and neutral ones, whereas congruent and neutral trials did not differ from one another. Since we measured the N2 component as the peak amplitude between 250 and 350 ms, this pattern of results implies that both physical and numerical size, as well as their relation to each other, has to be processed prior to this peak. Furthermore, enhancement in the N2 amplitude was apparent only in incongruent trials, when the irrelevant stimulus dimension (physical size) was misleading and interfered with the numerical size. It was not present in neutral and congruent trials, when physical size did not contradict numerical size.

As mentioned before, only a handful of papers have investigated the N2 component in the numerical Stroop task and their findings were inconclusive. Huang et al. [3] did not report any effects on the anterior N2 with adult participants. Yao et al. [12] found marginally more negative N2 on fronto-central electrodes in congruent than in incongruent and neutral trials in children after receiving mental-abacus training, but did not report any effects in the control group. Furthermore, the effects on the N2 component were also observed later in time, around 370–470 ms, than in the inhibition tasks. As opposed to Yao et al. [12] and Huang et al. [3], using the hybrid go/nogo numerical Stroop paradigm, we found a congruency effect on the fronto-central N2 component in adult participants. Furthermore, the timing and the pattern of the N2 component in the current study are in accord with those reported by hybrid Flanker go/nogo tasks. Namely, that incongruent trials elicit a more negative anterior N2 component than congruent trials (those tasks did not include neutral trials). Thus, it seems that when the irrelevant stimulus dimension induces interference, as in the case of incongruent trials, then this interference is inhibited between 250 and 350 ms. By contrast, when the irrelevant stimulus dimension does not induce interference, as in congruent and neutral trials, no evidence of interference inhibition was observed. At the same time, we also found no evidence for an interaction between physical size and numerical size on the P3 component, as it was not modulated by congruency effects.

Taken together, this pattern of results seems to support the early interaction account, namely, that physical and numerical size interact at the stage of perceptual processing. Furthermore, it also implies that the interference caused by misleading information of physical size in incongruent trials is probably resolved by inhibitory processes that occur very early, between 250 and 350 ms. Introducing the go/nogo condition was necessary to reliably elicit the inhibition-related N2 component and to obtain an unconfounded measure of inhibition, i.e., when the inhibition of an overt motor response is not confounded by the initiation of another, as in the classical numerical Stroop task. It is, however, still unclear how numerical distance affects the N2 component. Even though both Yao et al. [12] and Huang et al. [3] included more than one distance, they did not investigate how distance may affect the N2 component. In the current study, we included only number pairs with a numerical distance of five.

Concurrently manipulating numerical and physical size in this task made it possible to create neutral trials. Including neutral trials is common in the numerical Stroop task and serves to separate facilitation and interference effects by contrasting congruent trials and incongruent trials with neutral ones, respectively [9]. However, trial manipulations in go/nogo and Flanker tasks do not allow the inclusion of neutral trials. In order to examine how the introduced go/nogo manipulation affected facilitation and interference effects in our experiment, we implemented a mass univariate analysis and compared neutral trials with congruent and incongruent ones. The results showed that in the go condition, congruent trials were more positive than neutral ones on centro-parietal electrode sites around 325–350 ms. By contrast, in the nogo condition, incongruent trials were more negative than neutral ones around 360–420 ms, starting at the fronto-central electrode site and reaching the centro-parietal electrode sites as time progressed. These results show that interference and facilitation effects may be altered by differences in cognitive control processes required by go versus nogo trials. More specifically, the conventional peak analysis on the inhibition-related N2 component showed that in incongruent trials interference from physical size is inhibited. The mass univariate analysis advanced this result by showing that when incongruent nogo trials are compared to neutral nogo trials, a stronger and longer inhibitory process is induced. It is likely that the larger negativity in incongruent trials between 360 and 420 ms reflects an additional inhibitory process or that it reflects response inhibition, which the conventional peak analysis was not able to detect. In go trials an opposite effect was observed. Go trials do not require the activation of response inhibition, which seems to create an extenuating condition for the integration of physical and numerical size. Thus, we suggest that the larger positivity in the go congruent trials reflect a facilitatory effect induced by the congruent information provided by the physical size. In short, we found evidence for more pronounced inhibitory processes in nogo trials and evidence for facilitation from physical size in go trials. Including neutral trials and performing a mass univariate analysis was necessary to discover these effects, however, further research is required to systematically pinpoint the exact nature of these processes.

When looking at the categorisation-related P3 component in go trials with small and large numerical distances, we found that peak latencies were earlier for large compared to small numerical distances, and for congruent compared to neutral and incongruent trials. These findings are in line with Gebuis et al. [2], who showed numerical distance and congruency effects on the peak latency of the P3 amplitude. Their study, however, did not include neutral trials, so it was not possible to separate interference and facilitatory effects of physical size. Our results show that the interaction of physical and numerical size on the categorisation-related P3 component goes both ways. The P3 latency appears earlier for congruent than for neutral trials, and it also appears earlier for neutral trials than for incongruent trials. Furthermore, we could not observe any effect on the P3 peak amplitude, that Gebuis et al. [2] interpreted as a marker of cognitive load [51]. The lack of congruency and distance effects on the P3 peak amplitude is probably due to differences in the cognitive load requirements between the classical numerical Stroop and the current paradigm, as in the latter one no choice response was required from the participants.

It is important to note, that in the current literature it is still debated what effect trial frequency has on the N2 and P3 components. Even though some studies reported larger ERPs in nogo than in go trials, irrespective of trial frequency, the difference in amplitude decreased when, for example, go and nogo trials were equally frequent [27,52]. Other studies even found a reversed effect when nogo trials were more frequent than go trials [30,48]. As the role of the proportion of nogo trials is a matter of ongoing debate, and because we wanted to test a novel hybrid paradigm, we decided to alternate frequent go trials with infrequent nogo trials. The classical go/nogo ERP effects are the most likely to be found in this case. As follows, it is unclear whether the observed go/nogo ERP effects in the current study reflect response inhibition required in nogo versus go trials, or whether they reflect response conflict created by the unequal ratio of the go and nogo trials.

Furthermore, as mentioned before, recent electrophysiological studies on inhibition have focused on disentangling and reliably identifying neural markers specific to different types of inhibition. Such studies implement hybrid inhibition tasks and aim to link conceptually distinguishable inhibitory control functions, such as interference inhibition and response inhibition, to distinct electrophysiological signatures, such as the N2 and the P3. Xie et al. [25] designed a hybrid Flanker task to elucidate the neural distinction between three types of inhibition: response inhibition, cognitive inhibition (suppressing an irrelevant rule), and interference inhibition (suppressing an irrelevant stimulus). They proposed that the N2 is a marker of suppressing an irrelevant stimulus. Groom and Cragg [23] reached similar conclusions when they showed that the N2 is possibly a marker of response conflict which is created by congruent and incongruent stimulus features. One limitation of their study is, however, that they did not keep the overall frequency of the congruent and incongruent trials equal. Therefore, it was not possible to determine whether differences in the N2 amplitude reflect the unequal frequency or the different stimulus features between congruent and incongruent trials. We circumvented this problem by keeping the overall frequency of congruent, neutral, and incongruent trials equal throughout the experiment. Thus, the current findings on the N2 amplitude do not reflect differences in trial frequency across the congruency conditions.

Another advantage of the current study is that concurrently manipulating two stimulus features (numerical and physical size) made it possible to create neutral trials, and therefore to elucidate whether the N2 amplitude is differentially modulated by congruent and neutral trials. This is not possible in Flanker tasks and classical go/nogo tasks, because in these paradigms only one stimulus aspect is manipulated. In the current task, the similarity between congruent and neutral trials is that there is no conflict between physical and numerical size, even though in congruent trials both stimulus features are manipulated while in neutral trials only numerical size is manipulated. If the N2 is indeed a marker of conflict created by relevant and irrelevant stimulus features—as put forward by Groom and Cragg [23]—then more negative N2 is expected only in incongruent trials compared to congruent and neutral trials, while these latter two are not expected to differ from each other. The results of the current study nicely mimicked this arrangement, which provides additional evidence that the N2 reflects interference inhibition and is not modulated by facilitatory effects. Moreover, the same pattern of effects was found in go and nogo trials.

The current results have significant implications for the field of inhibition research, that investigates the exact mechanisms underlying the N2 component. Moreover, they show that the current paradigm may be a suitable method to investigate open questions about distinct neural markers of different inhibitory processes, as well as more specific ones. For example, in the field of numerical cognition, it could be used to disentangle how such inhibitory subprocesses contribute to numerical processing in normally developing children and children with mathematical learning disabilities. It is important to note, however, that the sample size was relatively small and therefore further research would benefit from replicating the current results and from addressing the possible modulatory effects of go/nogo frequency and numerical distance (see above).

Taken together, the current study investigated the electrophysiological correlates of the interaction between numerical and physical size in a modified go/nogo numerical Stroop task. The findings show that the interference between physical and numerical size is probably resolved by inhibitory processes, and that facilitatory effects may be affected by cognitive control processes required by go versus nogo trials.

## Data Availability

Data requests can be made to judit.pekar@fu-berlin.de.
